# A systemic lupus erythematosus patient with persistent elevated conjugated bilirubin as the initial symptom: A case report

**DOI:** 10.1097/MD.0000000000036999

**Published:** 2024-02-09

**Authors:** Jun Liu, Tingting Shen, Long Li, Xingyi Li, Fang Zhao, Xiaoxia Liu, Shan Zhang, Pengjia Wu, Na Li, Jiashun Zeng

**Affiliations:** a Department of Rheumatology and Immunology, The Affiliated Hospital of Guizhou Medical University, Guiyang, Guizhou, China; b Guizhou Provincial People’s Hospital, Guiyang, Guizhou Province, China; c Guizhou Medical University, Guiyang, Guizhou Province, China.

**Keywords:** case report, cholestasis, hyperbilirubinemia, lupus hepatitis, systemic lupus erythematosus

## Abstract

**Rationale:**

While some systemic lupus erythematosus (SLE) patients may experience varying degrees of liver function abnormalities, only a small portion of these cases have clinical significance, and the majority of patients typically exhibit low levels of serum bilirubin. However, in this article, we present a case of a middle-aged female patient with SLE who exhibited persistent skin jaundice as her initial symptom, offering a fresh perspective on diagnosing and treating patients who exhibit unexplained liver dysfunction and SLE combined with liver injury.

**Patient concerns:**

A 45-year-old woman was initially admitted to the hospital due to yellowing of the skin and sclera, and her symptoms did not improve significantly during treatment. The results were abnormal after relevant immunological tests.

**Diagnoses:**

Persistent non-conjugated bilirubin elevation due to lupus hepatitis.

**Interventions:**

The use of methylprednisolone sodium succinate (40 mg/Qd) and mycophenolate mofetil (0.75 g/d) suppressed immunity, polyolefin choline (20 mL/d) and glutathione (0.6 g/Qd) improved liver function, and nutritional support therapy.

**Outcomes:**

After 2 weeks of treatment, a significant decrease in the yellow skin and sclera of the patient was observed.

**Lessons:**

Most clinicians overlook that liver function abnormalities are the main manifestation of SLE, resulting in many patients not receiving timely treatment. This study highlights the importance that SLE is also a cause of abnormal liver function.

## 1. Introduction

Systemic lupus erythematosus (SLE) is a typical connective tissue disease with multiorgan involvement, often presenting clinically with erythema of the skin, glomerulonephritis, serositis, and blood and central nervous system involvement. SLE is usually treated with nonsteroidal anti-inflammatory drugs, adrenocortical hormones, and immunosuppressants. The most common causes of death from SLE are lupus nephritis, neuropsychiatric lupus, and multi-organ failure due to secondary infection.

Usually the liver is not the primary organ affected by SLE, so liver function abnormalities are not included in the diagnostic and classification criteria for SLE. Studies have shown that 25% to 50% of lupus patients have abnormal liver function indicators. There may be 3 reasons for the high incidence of abnormal liver function indicators in SLE patients: (1) liver parenchymal damage caused by SLE autoimmunity, commonly referred to as lupus hepatitis; (2) specific liver injury with autoimmune liver disease (AILD); (3) liver damage combined with other non-autoimmune liver diseases, such as drug-induced hepatitis, viral hepatitis.

In patients with SLE, lupus hepatitis is diagnosed in only 9.3% of patients. Lupus hepatitis occurs more frequently in active patients than in patients in remission. Most patients with lupus hepatitis present with mildly to moderately elevated serum aminotransferase levels, and in this case persistent cutaneous jaundice is the first symptom.

## 2. Case report

Four months ago, a 45-year-old Chinese female patient had yellow skin and sclera. The initial examination revealed elevated levels of transaminase and total bilirubin. Based on the patient’s clinical manifestations and relevant diagnostic test results, the diagnosis is acute hyperbilirubinemia and liver failure. During this period, the patient experienced recurrent episodes of the disease, and the treatment was mainly focused on providing supportive care to alleviate symptoms. A week ago, the patient visited the other hospital. The MRI of the upper abdomen revealed signs of the compensatory phase of cirrhosis and acute cholecystitis. The results of abdominal color ultrasound showed an uneven liver echo and a strong echo in the liver and gallbladder wall. Liver function test results showed: ALT 182 U/L, AST 369 U/L, TBiL 250 μmol/L, DBiL 185 μmol/L, IBiL 65 μmol/L, TBA 473.7 μmol/L. Anti-nuclear antibody (ANA) antibody panel test showed anti-nuclear antibody was positive, anti-nuclear antibody titer was positive.

The patient visited the Department of Hepatology of our hospital on October 11, 2022. The physical examination showed that the skin, mucous membrane, and sclera of the whole body were stained yellow, the capillaries on the face were dilated, and the spider nevi and liver palms appeared on the chest and neck. The patient reported a long history of alcohol consumption in the past. The equivalent alcohol intake was about 200 g/time. Liver function test results showed: ALT 181.70 U/L, AST 319.30 U/L, TBiL 242.41 μmo1/L, DBiL 197.62 μmol/L, IBil 44.79 μmol/L, TBA 544.60 μmol/L, hypersensitivity C-reactive protein 11.130 mg/L, IL-6 8.08 pg/mL, PCT 0.25 ng/mL, immunoglobulin IgG 24.90 g/L, complement C3 0.65 g/L, and complement C4 0.148 g/L. Urinary routine test indicated a positive result for urinary bilirubin. Five items of hepatitis B: anti-HBe > 4.20PEIU/mL, anti-HBc > 5.50 IU/mL. ANA antibody panel test (including anti-dsDNA) showed positive results for ANA at a titer of 1:1000, as well as positive results for anti-double-stranded DNA (anti-dsDNA) antibodies. Additionally, anti-ribosomal P-protein antibodies were suspected to be positive. The Coombs test was positive. There was no obvious abnormality in detecting highly sensitive B DNA, blood ammonia, and autoimmune liver. Chest and abdomen CT showed suspected liver cirrhosis, the uneven density of liver parenchyma, and cholecystitis sign. Upper abdominal MRI showed abnormal signal in liver parenchyma and cholecystitis. MR enhancement scan of upper abdomen showed large abnormal enhancement focus in the liver, hepatic sinus obstruction syndrome? Signs of cholecystitis. DWI of upper abdomen: slightly high signal in the liver. MR cholangiography showed gallbladder effusion and cholecystitis are considered. Color Doppler ultrasound of liver and portal vein system showed intrahepatic echo changes and intrahepatic patch echo areas; there were many strong echo spots in the liver, and the portal vein blood flow was smooth. After admission, the patient was treated with symptomatic support such as liver protection, anti-jaundice, enzyme lowering, and intestinal microflora regulation. The patient’s jaundice did not show significant improvement during hospitalization. The relevant immunologic tests were completed, and the results showed abnormalities. Considering the possibility of SLE, lupus liver, and biliary system impairment, the patient was transferred to our department for a liver function test, which revealed ALT 194.70 U/L, AST 487.40 U/L, TBiL 228.20 μmo1/L, DBiL 159.90 μmol/L, IBil 68.30 μmol/L, and TBA 372.77 μmo/L. The patient’s immunity was inhibited by methylprednisolone sodium succinate (40 mg/Qd) and mycophenolate mofetil (0.75 g/d); polyolefinylcholine (20 mL/d) and glutathione (0.6 g/Qd) improve liver function and promote bile excretion; The patients were also treated with nutritional support. Following the aforementioned treatment for 2 weeks, a significant reduction in the patient’s yellow skin and sclera was observed. The patient underwent a liver function test, which showed ALT 141.50 U/L, AST 72.00 U/L, TBiL87.50 μmol/L, DBiL 58.60 μmo1/L, IBil 28.90 μmol/L, TBA 86.43 μmol/L. The patient presented with an acute onset of the disease and significant liver function impairment, along with a significant elevation in TBA levels. The presence of positive anti-dsDNA and anti-nuclear antibodies was observed, but the absence of AMA and other autoimmune hepatitis (AIH)-related indicators led to the exclusion of AIH. The patient’s Coombs test was positive. According to the patient’s medical history, clinical manifestations, and relevant examinations, it was determined that the primary cause of the persistent unconjugated bilirubin elevation was lupus hepatitis.

## 3. Discussion

SLE is a heterogeneous disease characterized by chronic autoimmune dysfunction of both the innate and adaptive immune systems.^[1]^ However, the commonly used classification criteria for SLE diseases do not include liver function impairment and biliary tract involvement.^[2,3]^ According to the epidemiological survey,^[4]^ up to 60% of SLE patients may have abnormal liver function but often have no apparent clinical significance. Multiple studies have demonstrated a significant decrease in serum bilirubin levels among SLE/LN patients, which is closely related to disease activity.^[5]^ Atsushi Takahashi^[6]^ et al investigated SLE patients with liver function impairment and concluded that drugs, SLE, and fatty liver are the primary causes of liver function impairment in SLE patients. Currently, most clinicians overlook abnormal liver function as the primary manifestation of SLE, resulting in many patients not receiving timely treatment.^[7]^ According to previous reports,^[8]^ more than half of the 15 patients with abnormal liver function as their initial symptom were diagnosed several weeks or even years after treatment. They were more likely to be misdiagnosed as hepatitis or delayed SLE. Liver function impairment is often characterized by abnormal levels of liver enzymes and the changes in bilirubin and albumin levels.^[9]^ Jaundice is a recognizable “distress signal” that is visible to the naked eye, making it one of the common reasons that most patients seek medical attention.

For patients presenting with liver function impairment, it is important to consider liver diseases and hemolytic conditions, as well as autoimmune diseases. Ellen C Ebert et al The ANA antibody panel test of this female patient also showed that anti-rRNP was suspiciously positive. As early as 1993, there were literature reports that,^[10,11]^ SLE patients complicated with chronic active hepatitis were also anti-rRNP positive. Therefore, the positive result of anti-rRNP antibody may indicate an association with SLE-related hepatitis and AIH, in addition to its potential predictive value for neuropsychiatric symptoms in SLE patients. The presence of the anti-rRNP antibody suggests a possible association with hepatitis activity in immune-related diseases.^[12]^ In SLE patients who test positive for this antibody, it is important to consider whether the antibody is continuously expressed in the body and monitor changes in liver function levels.

Monocytes and macrophages destroy aging red blood cells in the circulation through hemolytic destruction by heme oxygenase and cathepsin.^[13,14]^ Later, uridine diphosphate glucuronic acid transferase 1A1 in the liver, combines free bilirubin with glucuronic acid to form binding bilirubin.^[15]^ Various metabolic substances in the human body are in a dynamic state of balance. The stable levels of various metabolic substances in the human body are maintained through a dynamic balance between their production and the consumption of excess metabolites.^[16]^ In this case, we reported a middle-aged female patient with a significant elevation of combined bilirubin. After further investigations, all abnormal indicators were found to be related to nonspecific digestive system symptoms. Recently, N Ferdous^[17]^ et al also reported a patient with severe lupus hepatitis. Unlike the patient in Ferdous report, our patient only presented with apparent jaundice. Through physical examination, we observed typical signs of cirrhosis, such as spider nevus and liver palm, but did not detect any symptoms of discomfort such as ascites. Biochemical indicators improve liver function impairment, but it is not accumulated kidney. Before the first visit, the patient did not take any medications, and the disease has a relatively short duration, mainly characterized by elevated levels of conjugated bilirubin; there are specific variations in the disease background and laboratory findings for this patient compared to others.

As we reported, for the female patient with lupus hepatitis, the liver impairment was mainly attributed to SLE. Bile is produced by hepatocytes and is closely linked to the liver cell metastasis function.^[18]^ However, when hepatocyte impairment occurs, it can cause bile production dysfunction. Bile cannot be transferred in time, leading to cholestasis, which increases the local oxidative stress of the liver and increases the level of heme oxygenase and bilirubin^[19]^; when the function and expression of intrahepatic transport proteins are disturbed, toxic bile acids will accumulate in the cells, thus aggravating the impairment of hepatocytes and creating a vicious cycle.^[20]^ Prolonged cholestasis can cause enlargement of the intrahepatic and extrahepatic bile ducts.

For SLE-related liver diseases, González Regueiro JA^[21]^ and others have emphasized the importance of firstly determining whether the liver injury is associated with SLE. First, SLE-related lupus hepatitis should be considered. Second, other autoimmune diseases (such as AIH, PBC, PSC, AIH-PBC/PBS) and alternative liver diseases (mainly including SLE combined with non-immune diseases, such as viral hepatitis, nonalcoholic hepatitis, drug-induced hepatitis, liver injury, biliary injury, vascular injury, and hemolysis-related disorders) should also be considered (Table 1).

**Table 1:** Characteristic of non-SLE-related autoimmune liver diseases.

Liver disease	Characteristics of clinical manifestations and biochemical indicators	Histological characteristics of liver
AIH PBC^[22]^ PBS Overlap syndrome (AIH-PBC/PBS)	Various clinical manifestations Characterized by hypergammaglobulinemia, positive circulating autoantibodies, histologic interface hepatitis, and liver function impairment.^[1,23]^ Type 1: ANA and/or anti-SMA: positive, type 2: anti-LKM-1 and/or anti-LC-1: positive.^[24]^ Fatigue, pruritus, skin pigmentation. Typical signs of cirrhosis, portal hypertension, and cholestasis. ALP: increased and lasted for more than 6 months, γ-GTP: increased, and total bilirubin: typically normal. AMA: positive. Chronic cholestatic liver disease, inflammation, and fibrosis lead to multifocal biliary stricture and progression to end-stage liver disease.^[22]^ ALP: increase Circulating autoantibody: Positive One of the subgroups of AIH mainly manifested in hepatitis and cholestasis serum liver tests and has the histological characteristics of AIH, PBC, or PSC.^[6,25]^ AST, ALT, ALP, GGT: increase AIH-PBC: AMA + ANA and/or ASMA AIH-PBS: P-ANCA ± ANA and/or ASMA	Interface hepatitis with lymphoplasmacytic infiltrates, hepatocellular rosette, emperipolesis, hepatocyte swelling or pyknotic necrosis.^[21]^ Florid duct lesion, Epithelioid granulomas, Ductopaenia, Feathery degeneration of liver cytoplasm. Onion skin fibrosis: inflammatory infiltration of intrahepatic and extrahepatic bile duct wall, with occlusive concentric peritubular loose fibrosis, Bile duct scar: degeneration and atrophy of bile duct cells, resulting in stricture and occlusion.^[5,26]^ AIH-PBC: Interface hepatitis + ductopenia AIH-PBS: Interface hepatitis + ductal impairment.

AIH-PBC = autoimmune hepatitis/primary biliary cirrhosis overlap syndrome, ANA = anti-nuclear antibody, anti-LC-1 = anti-liver cytosol type 1, anti-LKM-1 = anti-liver kidney type 1, anti-SMA = smooth muscle actin, PBC = primary biliary cholangitis, PSC = primary sclerosing cholangitis.

## 4. Conclusion

In summary, we report a case of SLE with lupus hepatitis and secondary biliary tract involvement. During the diagnosis and treatment of SLE, particularly in regions with a high prevalence of hepatitis B cirrhosis like China, it is important to exclude hepatitis B or alcohol-related liver injuries and also consider differential diagnosis from other autoimmune liver diseases. Liver biopsy and liver fibrosis examination are also necessary, especially for identifying unknown causes of liver dysfunction and cirrhosis. Cholestasis provides a more comprehensive diagnosis. Hormone therapy combined with immunosuppressive therapy is recommended for these patients. In this case, the combined treatment of methylprednisolone and mycophenolate has been implemented and has demonstrated favorable results.

## Author contributions

**Conceptualization:** Jun Liu, Long Li, Jiashun Zeng.

**Formal analysis:** Jun Liu, Tingting Shen, Xiaoxia Liu.

**Investigation:** Jun Liu, Shan Zhang, Pengjia Wu, Na Li.

**Methodology:** Long Li, Xingyi Li, Jiashun Zeng.

**Resources:** Jun Liu, Long Li, Shan Zhang, Pengjia Wu, Jiashun Zeng, Na Li.

**Supervision:** Jun Liu, Jiashun Zeng.

**Visualization:** Xingyi Li, Fang Zhao.

**Writing – original draft:** Xingyi Li, Fang Zhao.
